# The Oral Administration of *Lactobacillus delbrueckii* subsp. *lactis* 557 (LDL557) Ameliorates the Progression of Monosodium Iodoacetate-Induced Osteoarthritis

**DOI:** 10.3390/cimb46080530

**Published:** 2024-08-16

**Authors:** Li-Wen Huang, Tzu-Ching Huang, Yu-Chen Hu, Bau-Shan Hsieh, Jin-Seng Lin, Han-Yin Hsu, Chia-Chia Lee, Kee-Lung Chang

**Affiliations:** 1 Department of Medical Laboratory Science and Biotechnology, College of Health Sciences, Kaohsiung Medical University, Kaohsiung 807378, Taiwan; lewehu@cc.kmu.edu.tw; 2 Department of Biochemistry, School of Medicine, College of Medicine, Kaohsiung Medical University, Kaohsiung 807378, Taiwan; huangtavia@gmail.com (T.-C.H.); huyujena@gmail.com (Y.-C.H.); hsiehbs@gmail.com (B.-S.H.); 3 Graduate Institute of Medicine, College of Medicine, Kaohsiung Medical University, Kaohsiung 807378, Taiwan; 4 Culture Collection & Research Institute, SYNBIO TECH INC., Kaohsiung 821011, Taiwan; jslin@synbiotech.com.tw (J.-S.L.); hanin@synbiotech.com.tw (H.-Y.H.)

**Keywords:** osteoarthritis, *Lactobacillus delbrueckii*, probiotics, MMPs, inflammation

## Abstract

Low-grade body inflammation is a major cause of osteoarthritis (OA), a common joint disease. Gut dysbiosis may lead to systemic inflammation which can be prevented by probiotic administration. The *Lactobacillus delbrueckii* subsp. *lactis* 557 (LDL557) has been demonstrated to have beneficial effects for anti-inflammation. This study investigated the effects of LDL557 on OA progress using monosodium iodoacetate (MIA)-induced OA of rats. Live or heat-killed (HK)-LDL557 of a low or high dose was administrated for two weeks before MIA-induced OA, and then continuously administrated for another six weeks. After taking supplements for eight weeks, OA progress was analyzed. Results showed that MIA induced knee joint swelling, chondrocyte damage, and cartilage degradation, and supplementation with a high dose of LDL557 reduced MIA-induced knee joint swelling, chondrocyte damage, and cartilage degradation. Additionally, MIA increased serum levels of the matrix-degrading enzyme MMP-13, while a high dose of HK-LDL557 decreased it for the controls. Simultaneously, bone turnover markers and inflammatory cytokines of serum were assayed, but no significant differences were found except for a TNF-α decrease from a low dose of live LDL557. These results demonstrated that supplementation with high doses of live LDL557 or HK-LDL557 can reduce the progression of MIA-induced OA in rats.

## 1. Introduction

Osteoarthritis (OA) is a prevalent arthritis type, posing a significant challenge to elderly healthcare. Symptoms like joint space narrowing, osteophyte hyperplasia, subchondral sclerosis, and cyst formation are detectable through radiographs, ultrasounds, and magnetic resonance imaging (MRI) [1]. OA affects the musculoskeletal system, causing joint failure, pain, and disability [2], potentially leading to reduced work capacity [3], mental health decline [4], and increased mortality [5]. OA is well known as an age-related degenerative disease. Additionally, recent research suggests that it is a low-grade inflammatory systemic disease that triggers metabolic abnormalities in chondrocytes, the resident cells responsible for maintaining joint homeostasis in articular cartilage [6]. Chondrocytes control the synthesis and degradation of extracellular matrix (ECM) components, including proteoglycans (aggrecan) and collagen. Notably, osteoarthritic joints have increased pro-inflammatory cytokine levels such as interleukin (IL) 1-β, IL-6, and tumor necrosis factor (TNF)-α [7,8], and ECM degradative enzymes such as matrix metalloproteinases (MMPs) and aggrecanase, a disintegrin and metalloproteinase with thrombospondin motifs (ADAMTSs), are active, which may cause inflammation of all joint tissues including the cartilage, synovial membrane, subchondral bone, and ligaments, and finally result in cartilage destruction [9,10]. Despite the severe impact of OA on health, there is still no convenient and effective treatment for OA currently [11].

Low-grade systemic inflammation is widely acknowledged as a critical factor in the development and progression of OA [12]. Gut dysbiosis, an imbalance in the gut microbiota, may lead to inflammation [13,14]. It is believed to be influenced by various factors such as age, gender, genetics, obesity, and overuse of antibiotics, which can affect inflammatory conditions and the progression of osteoarthritis [15]. In recent years, the concept of the “gut-joint axis” has emerged which proposes that a change in the balance of intestinal flora may regulate immune and inflammation status which will improve joint health. As is well known, the colon is mainly composed of bacterial flora, including three phyla: *Bacteroidetes*, *Firmicutes*, and *Actinobacteria* [16]. Moreover, reports indicate that the abundance of *Lactobacillus* spp. is lower in OA patients compared to healthy individuals, while the number of *Clostridiales* orders is higher [17]. Certain intakes of probiotics like *Lactobacillus acidophilus* [18,19], *Lactobacillus paracasei* subsp. *paracasei* M5 [20], or *Clostridium butyricum* [21] may lessen OA symptoms in experimental rat or murine OA models. Therefore, interventions that target the gut microbiota, such as the use of probiotics or prebiotics, could be a viable option for both preventing and treating OA.

*Lactobacillus delbrueckii* subsp. *lactis* 557 (LDL557) is a species classified as having a biosafety level of 1 by the American Type Cell Collection (ATCC) and complies with the European Union’s Qualified Presumption of Safety (QPS) standards for use in food and feed [22]. In addition, its use as a food ingredient has been approved by Taiwan’s Ministry of Health and Welfare. Monosodium iodoacetate (MIA), an inhibitor of glyceraldehyde-3-phosphate dehydrogenase, can cause chondrocyte death when injected into the articular cartilage of rodents [23]. This MIA-induced OA model is considered appropriate for studying human OA, since it may induce chondrocyte death and articular cartilage loss which are similar to those observed in human OA [23,24]. Accordingly, this study aimed to evaluate the effectiveness of LDL557 on OA progression in an MIA-induced OA model of rats. In addition to exploring live LDL557 supplementation, we also tested whether its metabolites were also effective in combatting OA progression in which LDL557 was treated by heat, called heat-killed (HK) LDL557 in this study. Herein, the MIA-induced OA rats had two weeks of pre- and six weeks of post-administration of low or high doses of live or HK-LDL557; then, joint integrity, biomarkers of bone turnover, and the expressions of pro-inflammatory cytokines and MMPs were analyzed. These results may provide insights into the possibility of using the LDL557 species or its metabolites to prevent OA progress.

## 2. Materials and Methods

### 2.1. Animals

Forty-eight male Sprague-Dawley rats, aged 6 weeks, were obtained from BioLASCO Taiwan Co., Ltd. (Charles River Technology, Taipei, Taiwan). They were kept in a controlled environment with a 12/12 h, light/dark cycle, and a temperature of 23 ± 1 °C. The rats were housed in groups of three per cage and given standard rodent chow (Altromin, Lage, Germany) for ad libitum feeding. The animal research conducted in this study complied with the ARRIVE guidelines [25] and was performed according to the 8th edition of the National Research Council (US) Committee Guide for the Care and Use of Laboratory Animals (https://www.ncbi.nlm.nih.gov/books/NBK54050/ (accessed on 27 January 2011)). The rats were assigned to different groups randomly, as described below. The Institutional Animal Care and Use Committee (IACUC) of Kaohsiung Medical University reviewed and approved the animal use protocol (Approval No. 111005; Approval date: 25 July 2022).

### 2.2. Sample Preparation

LDL557 was originally isolated from corn by SYNBIO TECH INC. (Kaohsiung, Taiwan), and identified as *Lactobacillus delbrueckii* subsp. *lactis* (detailed information is in Supplementary Figure S1). LDL557 was cultured in an MRS broth (Difco Corp., Sparks, MD, USA) at 37 °C without shaking for 16 h, and then bacterial cell pellets were collected by centrifugation and spray-drying. Heat-killed LDL557 (HK-LDL557) cells were prepared under the same culture condition as LDL557, but the liquid bacterial culture was additionally heated at 85 °C for 30 min before undergoing the centrifugation and spray-drying procedures. The cell number of HK-LDL557 was quantified by using a Quantom Tx Microbial Cell Counter with the Quantom total cell staining kit (Logos Biosystem, Anyang, Republic of Korea) following the manufacturer’s instructions and in brief, 10 μL of diluted LDL557 samples was mixed with 1 μL of the cell staining dye, 1 μL of cell the staining enhancer, and 8 μL of the loading buffer. Afterward, 6 μL of the mixture was placed onto a Quantom M50 cell counting slide and centrifuged at 300 RCF for 10 min using a Quantom Centrifuge (Logos Biosystem, Anyang, Republic of Korea). The parameters for the Quantom Tx Microbial Cell Counter were as follows: light intensity, level 1; size gating, ~0.3 to 50 µm; roundness, 0%; de-clustering level, 10; and detection sensitivity, 9.

### 2.3. Experimental Design

The rats were randomly divided into six groups and each group had eight rats. The groups consisted of a control sham group, an MIA-induced OA group, and four groups of MIA-induced OA receiving once-daily LDL557 supplementation of a low dose (1.03 × 10^9^ cfu/kg BW rat) or a high dose (5.14 × 10^9^ cfu/kg BW rat) with live or heat-killed (HK) LDL557 in a water solution. The low dose of LDL557 given to rats was calculated based on the dose of 1 × 10^10^ cfu/day for humans and followed by an adjustment of the differences in body surface area between species [26], while the high dose of LDL557 was five times the low dose. At the start, the supplementation groups took LDL557 by gavage for two weeks because it imitated the scenario of preventive supplement intake before the disease occurs. Then, OA was induced by intra-articular injection of MIA (1 mg/20 μL of 0.9% sterile saline) into left knee joints using a 31-gauge needle, while the sham group was injected with 0.9% sterile saline as previously described [27]. The OA experimental supplementation groups were continuously orally administered LDL557 by gavage for another six weeks. Body weight and joint swelling were monitored weekly during the experimental periods. At the end of eight weeks, the rats were euthanized using CO_2_ then the serum and left leg were collected. All samples were stored at −80 °C until further analysis.

### 2.4. Preparing Frozen Sections of Rat Knee Joints

The femur of the rat was fixed in a 10% formalin buffer for 72 h, followed by decalcification in a 10% formic acid solution at room temperature for another 72 h [28]. Each joint was then embedded in an optimal cutting temperature compound (O.C.T) and stored at −80 °C until analysis. For the analysis, the femur was sectioned sagittally into a 5 μm thicknesses by a Leica CM1950 cryostat (Leica Biosystems, Nussloch, Germany) and stained with H&E or Safranin O/Fast Green (Sigma-Aldrich, St. Louis, MO, USA).

### 2.5. Hematoxylin-Eosin (H&E) Staining

To analyze the articular cartilage structure, hematoxylin stained the nuclei blue-purple, and eosin stained the cytoplasm pink-red. The tissue was stained with 37% hematoxylin (Sigma-Aldrich Co., LLC., St. Louis, MO, USA) for 2 min, 1% eosin (Sigma-Aldrich Co., LLC., St. Louis, MO, USA) for 5 min, and dehydrated using increasing alcohol concentrations. Observations were made under an Olympus CKX41 microscope (Shinjuku, Tokyo, Japan). The modified Mankin scoring system evaluated joint damage and recovery, which rates damage from 0 (normal) to 6 (severe), with higher scores indicating more severe chondrocyte damage [29].

### 2.6. Safranin O/Fast Green Staining

To detect acidic proteoglycans in cartilage, Safranin O was used to stain the cartilage tissue orange-red, and Fast Green was used stain the other tissues green. The tissue was stained with 0.001% Fast Green (Sigma-Aldrich Co., LLC., St. Louis, MO, USA) for 5 min, followed by 1% acetic acid for 10–15 s, then with 0.1% Safranin O (Sigma-Aldrich Co., LLC., St. Louis, MO, USA) for 5 min. The tissue was dehydrated using increasing alcohol concentrations and inspected under an Olympus CKX41 microscope (Shinjuku, Tokyo, Japan). Joint damage was assessed using OARSI scoring, which grades cartilage matrix loss from 0 (normal) to 6 (severe) and cartilage level damage from 0 to 4. Scores are multiplied, resulting in scores ranging from 0 to 24, with higher scores indicating more severe damage [30].

### 2.7. Measurement of Serum Biomarkers in Rats

After sacrifice, rat serum was obtained by centrifuging the blood samples at 3000× *g* for 15 min, then the resulting serum samples were divided into aliquots and frozen at −80 °C until further use. There was no repeated freezing or thawing of specimens before obtaining measurements. Sandwich enzyme-linked immunosorbent assay (ELISA) kits were used to detect the serum levels of cytokines TNF-α (cat. no. MBS700574), IL-1β (cat. no. MBS2125216), IL-6 (cat. no. MBS2707980), and IL-10 (cat. no. MBS2104743) (MyBioSource, San Diego, CA, USA), bone turnover biomarkers of CTX-I (cat. no. MBS458687) and PINP (cat. no. MBS4501851) (MyBioSource, San Diego, CA, USA), and matrix-degrading enzymes of MMP-1 (cat. no. MBS2701169), MMP-3 (cat. no. MBS2701197), and MMP-13 (cat. no. MBS2701183) (MyBioSource, San Diego, CA, USA). The ELISA procedure was strictly performed according to the manufacturer’s instructions. A total of 100 μL of the sample was added to each well and incubated for 1 h. Then, the primary antibody reagent was incubated for an additional 1 h. Next, the secondary antibody reagent was incubated for 30 min. After that, the substrate solution was added and incubated for 10–20 min. Finally, the stop solution was added and the results were read at 450 nm. All samples were examined in duplicate within each assay.

### 2.8. Statistical Analysis

All data are presented as means ± standard deviation (S.D.) and were analyzed using a one-way ANOVA with a post-hoc Tukey’s test. All statistical analyses were performed using GraphPad Prism 8.1.1 (GraphPad Software, San Diego, CA, USA). A *p*-value < 0.05 was considered statistically significant. The actual numerical information of the 95% CI and *p*-value between each group is shown in Supplementary Figures S2 and S3.

## 3. Results

### 3.1. Effect of LDL557 on Body Weight and Joint Swelling

**Figure 1:**
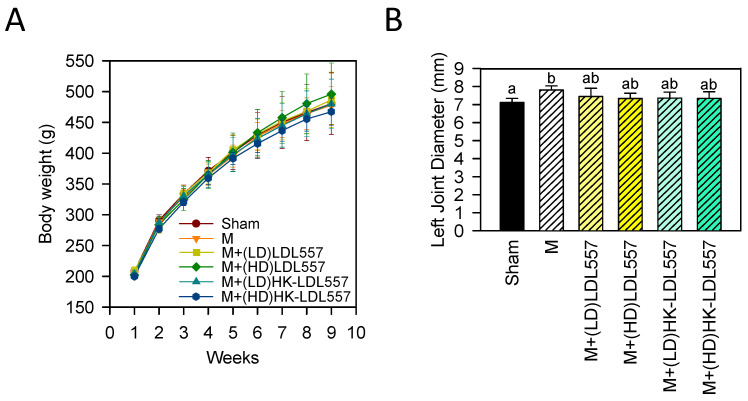
Effect of live or HK-LDL557 on (**A**) body weight and (**B**) left knee joint diameter in monosodium iodoacetate (MIA)-induced osteoarthritis (OA) rats. The results are presented as means ± S.D. (*n* = 8). Sham: control; M: MIA; LD: low dose; HD: high dose; HK: heat-killed; and LDL557: *Lactobacillus delbrueckii* subsp. *lactis* 557. Statistical differences were analyzed using one-way ANOVA with post-hoc Tukey’s test. Different letters indicate significant differences among groups (*p* < 0.05).

After two weeks of pretreatment followed by six weeks of post-treatment of low or high doses of live or HK-LDL557, there was no statistically significant difference in body weight among groups (Figure 1A); even the MIA-induced group was not different from the sham group. As Figure 1B shows, the left knee diameter of the MIA-injected group was markedly increased as compared to the sham group, whereas each group of LDL557 supplementation was not significantly different from the sham or MIA-induced group in knee joint diameter.

### 3.2. Effect of LDL557 on Knee-Joint Histopathologic Changes

**Figure 2:**
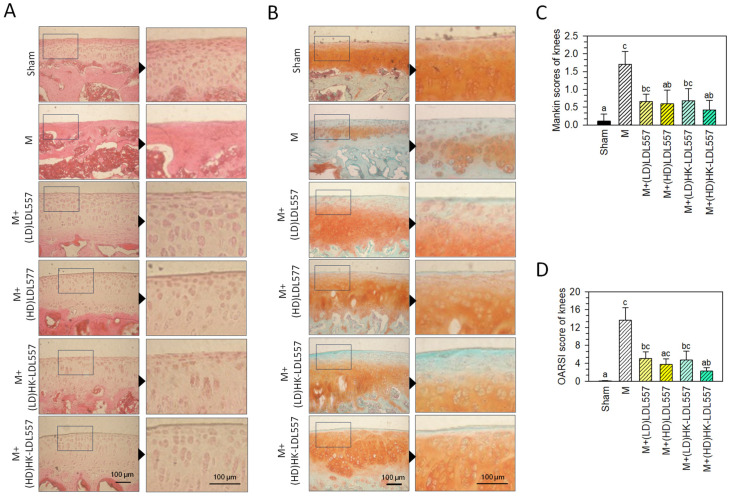
Effects of live or HK-LDL557 with a low or high dose on the morphology and histology of the articular cartilage of MIA-induced OA rats. Photomicrographs of histomorphological changes in joint cartilage stained with (**A**) H&E or (**B**) Safranin O/Fast Green. Inset boxes in left panels indicate higher magnification images in right panels. The evaluation was conducted using (**C**) the modified Mankin scoring system or (**D**) the OARSI scoring system, respectively. The results are presented as means ± S.D. (*n* = 8). Statistical differences were analyzed using one-way ANOVA with post-hoc Tukey’s test. Different letters indicate significant differences among groups (*p* < 0.05).

Figure 2A,C shows H&E-stained knee joints and modified Mankin histological scores for each rat group. The untreated sham group exhibited smooth, clear, and complete cartilages with regularly arranged chondrocytes. The MIA-induced group without supplementation showed irregular surfaces, cartilage tissue loss, and degraded articular cartilage with missing chondrocytes. Live or HK-LDL557 supplementation tended to lower Mankin scores, with high doses reaching statistical significance, resembling the sham group. Figure 2B,D presents Safranin O/Fast Green-stained knee joints and OARSI scores. The sham group had uniform Safranin O staining (red), while the MIA-induced group had cartilage damage with reduced Safranin O and increased Fast Green staining (green), indicating proteoglycan loss. High-dose HK-LDL557 supplementation significantly decreased OARSI scores. Supplementary Figures S4 and S5 provide additional cartilage images.

### 3.3. Effect of LDL557 on the Biomarkers of Matrix-Degrading Enzymes

**Figure 3:**
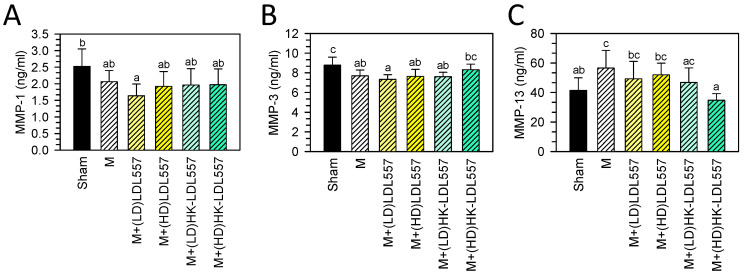
Effects of live or HK-LDL557 with low or high doses on serum matrix-degrading enzymes. (**A**) MMP-1, (**B**) MMP-3, and (**C**) MMP-13 were analyzed. The results are presented as means ± S.D. (*n* = 8). Statistical differences were analyzed using one-way ANOVA with post-hoc Tukey’s test. Different letters indicate significant differences among groups (*p* < 0.05).

Past studies have shown the serum MMP levels of patients with OA are altered [31,32,33]. Therefore, after eight weeks of LDL557 administration in MIA-induced OA rats, serum MMP-1, MMP-3, and MMP-13 levels were analyzed to evaluate the extent of cartilage degradation. It was found that the MIA injection group did not exhibit a change in MMP-1 levels (Figure 3A), but exhibited a decrease in MMP-3 (Figure 3B) and an increase in MMP-13 (Figure 3C) as compared with the sham group. The groups for live or HK-LDL557 supplementation with low or high doses were not different from the MIA-induced group in serum MMP-1 and MMP-3 levels. However, a high dose of HK-LDL557 effectively reduced MMP-13 expression (Figure 3C).

### 3.4. Effect of LDL557 on the Biomarkers of Bone Turnover

Experimental and clinical studies have established that the structural integrity of articular cartilage depends on normal subchondral bone turnover. In general, biochemical markers of bone turnover are classified into bone resorption and bone formation markers. According to research, the serum procollagen type I N propeptide (PINP) is connected with bone and osteophyte formation [34]. On the other hand, the serum C-terminal telopeptide of type I collagen (CTX-I) is linked with bone resorption [35,36].

**Figure 4:**
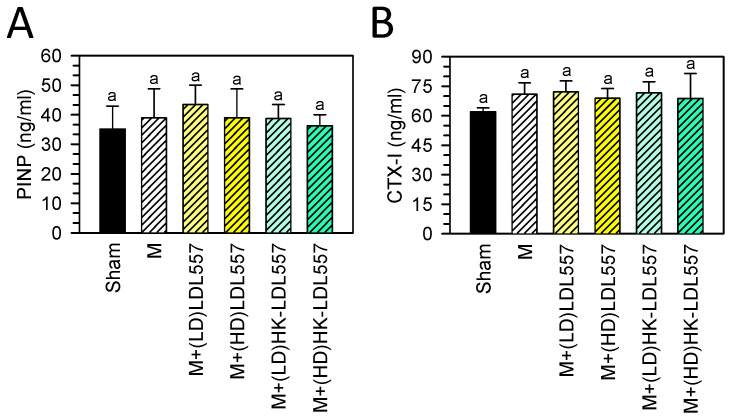
Effects of live or HK-LDL557 with low or high doses on serum bone turnover biomarkers. (**A**) PINP and (**B**) CTX-I were analyzed. The results are presented as means ± S.D. (*n* = 8). Statistical differences were analyzed using one-way ANOVA with post-hoc Tukey’s test. Different letters indicate significant differences among groups (*p* < 0.05).

After supplementation for eight weeks, the concentrations of bone turnover biomarkers, namely PINP and CTX-I, were measured, as shown in Figure 4. The results showed that neither MIA injection nor supplementation with LDL557 caused any significant changes in the levels of PINP and CTX-I in serum.

### 3.5. Effect of LDL557 on the Expressions of Inflammatory Cytokines

**Figure 5:**
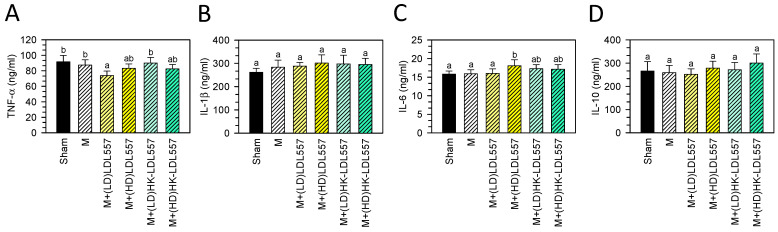
Effects of live or HK-LDL557 on serum cytokine levels in MIA-induced OA rats. (**A**) TNF-α, (**B**) IL-1β, (**C**) IL-6, and (**D**) IL-10 were analyzed. The results are presented as means ± S.D. (*n* = 8). Statistical differences were analyzed using one-way ANOVA with post-hoc Tukey’s test. Different letters indicate significant differences among groups (*p* < 0.05).

After eight weeks of LDL557 administration in MIA-induced OA rats, serum TNF-α, IL-1β, IL-6, and IL-10 levels were measured to evaluate inflammation status. The MIA injection group did not exhibit a change in levels of TNF-α, IL-1β, IL-6, or IL-10 (Figure 5A–D) as compared to the sham group. Groups receiving a low dose of live LDL557 supplementation showed reduced TNF-α levels compared with the MIA or sham group. A high dose of live LDL557 also increased IL-6 levels compared to the MIA or sham group.

## 4. Discussion

Up to now, no report has investigated the effects of LDL557 on the prevention or treatment of OA. This study first showed supplementation of LDL557 can ameliorate OA progression in an MIA-induced OA rat model. LDL557 was supplemented in live or heat-killed forms at a low (1.03 × 10^9^ cfu/kg BW rat) or high dose (5.14 × 10^9^ cfu/kg BW rat) for eight weeks. According to the assessment of joint damage severity or ECM loss measured by the modified Mankin scoring system or the OARSI scoring system (Figure 2), supplementation with both live and HK-LDL557 reduced cartilage tissue loss and articular cartilage degradation. Among them, a high dose of live or HK-LDL557 supplements had better effects. However, the optimal LDL557 supplementation dosage still requires further study since live LDL557 supplementation can significantly reduce MMP-1, MMP-3, and pro-inflammatory cytokine TNF-α levels only at low doses, not high doses (Figure 2 and Figure 5). A high dose of HK-LDL557 effectively reduced MIA-induced OA progression by alleviating joint damage and ECM loss (Figure 2), and reduced MMP-13 expression (Figure 3). In addition, this may be related to the fact that live and HK-LDL557 affect the balance of the gut microbiome and its metabolites to reduce systemic inflammation. However, further research is needed to confirm this effect.

It is well known that OA is linked to increased levels of inflammatory cytokines, particularly IL-1β and TNF-α, which can cause a loss of the ECM by upregulating the production of MMPs such as MMP-1, MMP-3, and MMP-13 [37,38,39]. Alternatively, these cytokines can directly inhibit the synthesis of type II collagen, aggrecan, and proteoglycans [40,41,42]. Additionally, these cytokines can lead to the production of other inflammatory cytokines like IL-6 [43,44]. IL-6 can further stimulate the production of MMP-1, MMP-3, and MMP-13 while reducing the expression of collagen type II [45,46]. Several studies have confirmed that inhibitors targeting pro-inflammatory cytokines such as IL-1β, IL-6, and TNF-α may relieve OA-related pain and decelerate the progression of OA [47,48,49]. On the other hand, anti-inflammatory medications like non-steroidal anti-inflammatory drugs (NSAIDs) can alleviate symptoms associated with OA [50]. Although NSAIDs are commonly used to relieve inflammation due to their analgesic and anti-inflammatory properties, their adverse effects, such as cardiovascular, renal and nervous system effects and gastrointestinal tract toxicity, are significant concerns [51]. Our results showed that the LDL557 supplement is effective in decreasing MIA-induced damage to knee joints in rats. However, this study does not demonstrate that LDL557 affects inflammatory cytokines expressions, except for TNF-α. This point will be further explored and clarified when the optimal dose of LDL557 is inspected.

In recent years, numerous studies have confirmed the potential health benefits of probiotics in treating various diseases, including gastrointestinal issues, obesity, anxiety, depression, autoimmune diseases, and allergies [52]. However, despite their usefulness, some risks are associated with their use in medical care. Reports showed that live probiotics may migrate from the intestinal lumen to extraintestinal sites (e.g., the bloodstream) and cause systemic infection in immunocompromised individuals [53,54], whereas inactivated probiotics may reduce this risk [53]. Our findings suggest that HK-LDL557 had equivalent or even more pronounced effects than live LDL557 in our model. We propose that there may be factors in HK-LDL557 such as their metabolites which contribute to its effects, and this merits further investigation. The use of HK-LDL557 as a nutritional supplement for joint health could be a safer and more beneficial option, particularly for patients who are immunocompromised.

Inflammation is widely recognized as a significant contributor to the development and progression of OA; moreover, many factors can worsen inflammation status including age, gender, genetics, obesity, and antibiotic overuse [15]. These factors can lead to an imbalance in gut bacteria, causing intestinal inflammation. Inflammation in the gut can increase the permeability of the intestinal barrier, also known as “leaky gut”, and lead to the production of inflammatory factors like IL-6, IL-8, and TNF-a [55,56], as well as harmful microbial products such as lipopolysaccharide (LPS) [57], which can enter into blood circulation and induce systematic inflammation [58], which is considered crucial in the development of OA and has led to the concept of the “gut-joint axis” [59]. Preclinical data, using 16S rRNA amplification to detect the relationship between gut microbiota and OA in patients and experimental animals with OA, found that the distribution of bacterial genera such as *Lactobacillus*, *Bifidobacterium*, *Clostridium*, *Streptococcus*, and *Bacteroidetes* may be a key factor [60]. *Lactobacillus* and *Bifidobacterium* have been the most extensively studied among these bacterial genera. Specific strains such as *Lactobacillus acidophilus* [18,19], *Lactobacillus paracasei* [20], *Lactobacillus rhamnosus* [61], *Lactobacillus plantarum* [62], *Lactobacillus casei* [63], and *Bifidobacterium longum* [64,65] have been highlighted in research. Studies indicate that these strains can significantly alleviate symptoms of OA in experimental rat or murine OA models by improving intestinal damage and inflammation, reducing systematic inflammatory factors, minimizing cartilage damage, and decreasing pain. According to the gut–joint axis theory, altering gut microbiota to inhibit chronic or systemic low-grade inflammation is proposed as a novel nutraceutical treatment for osteoarthritis [17,66]. Herein, the efficacy of LDL557 on joint health has been demonstrated for the first time.

There are limitations in this study, which are (1) the concerns that the MIA-induced OA model in rats is not a naturally occurring disease. Mice and rats are commonly used for OA studies, and different approaches are used to induce the condition, including mechanical, surgical, and chemical methods [67]. Among these methods, the mechanical model is the most commonly used as it closely mimics the OA process. However, it requires specialized equipment such as a material testing machine (InstronElectroPulse E1000, Instron, Norwood, MA, USA) [68]. Although surgical or chemical approaches have their limitations, MIA-induced osteoarthritis of the rat knee is still a valuable model for chemical-related studies. It is less invasive than surgical models and induces cartilage damage, loss of the proteoglycan matrix, and stiffness resembling human osteoarthritis [24]. This model also allows for measurable changes in joint motion, tactile allodynia, progressive radiographic degeneration, and microscopic inflammation of the synovium [69]. (2) The different kinds of gut microbiota were not evaluated before or after LDL557 supplementation; (3) the optimal dose of LDL557 was not evaluated; and (4) the differences between effective components in HK-LDL557 and live LDL557 are not yet approved. Overall, this study provides clues for LDL557 application; however, the points of what are the effective metabolites, what are the optimal ranges of LDL557 supplementation, and what are the impacts on the composition of the gut microbiome, need to be further verified.

## 5. Conclusions

This study demonstrates that supplementation with high doses of live or HK-LDL557 can lessen knee joint swelling, chondrocyte damage, and cartilage degradation in rats with MIA-induced OA. This indicates that LDL557 may ameliorate the severity of OA in an MIA-induced rat model. LDL557 could be used as a nutritional supplement for joint health.

## Data Availability

Data are contained within the article and the Supplementary Materials.

## Supplementary Materials

The following supporting information can be downloaded at: https://www.mdpi.com/article/10.3390/cimb46080530/s1, Figure S1: Bacterial strain LDL557 (STCC0557) was identified by using multilocus sequence analysis (MLSA); Figure S2: The actual numerical information of the 95% CI between each group; Figure S3: The actual numerical information of the *p*-values between each group; Figure S4: Effects of live or HK-LDL557 with low or high dose on the morphology of the MIA-induced OA rat’s articular cartilage (H&E stain); Figure S5: Effects of live or HK-LDL557 with low or high dose on the histology of the MIA-induced OA rat’s articular cartilage (safranin O/fast green).
