# Supplementary material for: The Oral Administration of Lactobacillus delbrueckii subsp. lactis 557 (LDL557) Ameliorates the Progression of Monosodium Iodoacetate-Induced Osteoarthritis
Source: Curr Issues Mol Biol. 2024 Aug 16;46(8):8969–80. doi: 10.3390/cimb46080530 (PMC11352892; doi:10.3390/cimb46080530)

Supplementary Figures S1(A)-(B)

Bacterial strain LDL557 (STCC0557) was identified as *Lactobacillus delbrueckii* subsp. *lactis* by using multilocus sequence analysis (MLSA). (A) The phylogenetic tree of LDL557 and *Lactobacillus delbrueckii* type strains based on 16S rDNA, fusA、gyrB、hsp60、ileS、pyrG、recA、recG gene sequences. (B) The percentage of sequence identity between LDL557 and *Lactobacillus delbrueckii* type strains.

(A)

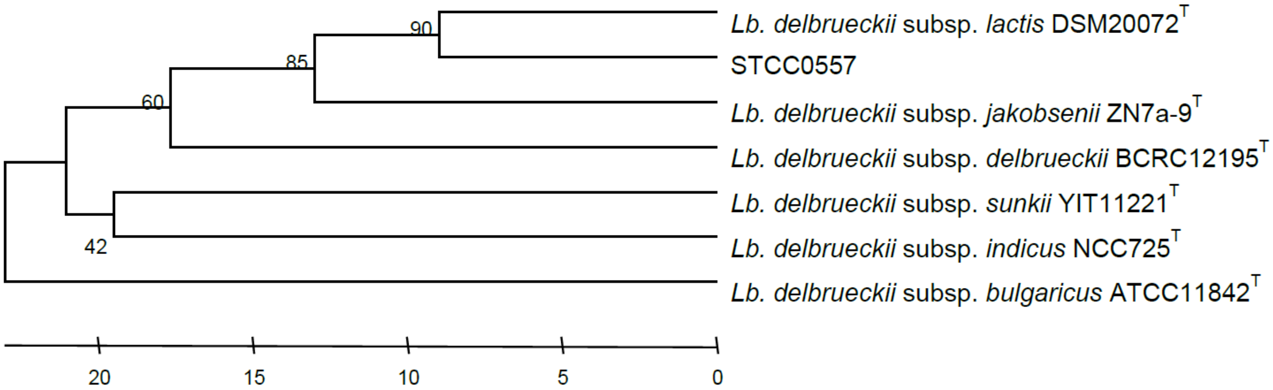

(B)

		1	2	3	4	5	6	7
1	*Lb. delbrueckii* subsp. *bulgaricus* ATCC11842^T	***	99	99.1	99.2	99.1	99.1	99.2
2	*Lb. delbrueckii* subsp. *delbrueckii* BCRC12195^T		***	99.1	99.3	99	99.2	99.4
3	*Lb. delbrueckii* subsp. *sunkii* YIT11221^T			***	99.3	99.2	99.2	99.3
4	*Lb. delbrueckii* subsp. *lactis* DSM20072^T				***	99.3	99.5	99.7
5	*Lb. delbrueckii* subsp. *indicus* NCC725^T					***	99.1	99.2
6	*Lb. delbrueckii* subsp. *jakobsenii* ZN7a-9^T						***	99.5
7	STCC0557							***

Supplementary Figures S2(A)-(E)
The actual numerical information of the 95% CI between each group is as shown in the table.

(A)

	95% CI of body weight (weeks)								
	1	2	3	4	5	6	7	8	9
Sham	203.0–213.3	283.3–298.4	321.8–345.9	353.0–389.7	378.0–424.9	398.5–460.5	414.7–485.6	427.9–503.6	438.7–522.3
M	201.0–208.8	281.3–296.0	317.9–339.4	350.1–379.4	380.3–410.2	408.2–445.9	428.9–466.1	442.9–485.6	451.8–503.7
M+(LD)LDL	203.9–214.5	272.2–293.1	323.0–344.7	354.0–382.3	385.9–424.7	410.4–451.3	426.5–477.0	437.1–498.7	449.0–524.3
M+(HD)LDL	199.1–205.5	273.8–294.1	309.8–339.5	346.5–384.5	375.0–427.6	401.7–464.8	422.3–493.2	440.3–520.7	453.9–538.1
M+(LD)HK-LDL	199.6–207.9	278.8–296.3	318.3–343.0	347.9–382.6	375.0–420.6	396.3–450.9	415.5–474.5	432.8–495.0	446.3–513.7
M+(HD)HK-LDL	196.1–203.5	272.4–281.7	315.9–325.4	350.8–367.9	383.3–399.7	403.7–427.5	422.2–451.0	439.1–472.2	448.9–485.8

(B)

	95% CI		
	Left knee joint diameter	Mankin score	OARSI score
Sham	6.9–7.3	0–0.1	0–0.2
M	7.6–8.0	1.4–2.0	9.3–18.0
M+(LD)LDL	7.1–7.8	0.5–0.8	2.7–7.5
M+(HD)LDL	7.1–7.6	0.3–0.9	1.7–5.8
M+(LD)HK-LDL	7.1–7.6	0.4–1.0	1.4–8.1
M+(HD)HK-LDL	7.0–7.7	0.2–0.4	1.2–2.4

(C)

	95% CI		
	MMP-1	MMP-3	MMP-13
Sham	2.1–3.0	8.1–9.5	34.3–48.6
M	1.8–2.3	7.2–8.2	46.5–66.6
M+(LD)LDL	1.3–1.9	6.9–7.7	39.3–59.2
M+(HD)LDL	1.6–2.3	7.1–8.2	45.3–58.6
M+(LD)HK-LDL	1.5–2.4	7.2–8.0	38.5–55.0
M+(HD)HK-LDL	1.6–2.4	7.8–8.8	31.0–38.5

(D)

	95% CI	
	PINP	CTX-I
Sham	28.6–41.7	60.5–63.7
M	30.7–47.2	66.1–75.8
M+(LD)LDL	38.0–48.9	67.4–76.8
M+(HD)LDL	30.8–47.2	64.7–73.1
M+(LD)HK-LDL	34.7–42.7	66.8–76.4
M+(HD)HK-LDL	33.1–39.4	58.1–79.4

(E)

	95% CI			
	TNF-α	IL-1β	IL-6	IL-10
Sham	84.6–98.5	248.1–275.1	15.1–16.5	231.7–299.9
M	82.0–93.1	257.5–309.2	15.0–16.8	233.5–285.3
M+(LD)LDL	69.0–78.7	275.9–300.0	15.0–17.0	232.2–271.8
M+(HD)LDL	78.6–88.0	271.9–330.7	16.7–19.4	254.3–303.5
M+(LD)HK-LDL	84.0–96.0	266.4–329.1	16.4–18.2	245.4–297.9
M+(HD)HK-LDL	77.7–87.1	273.6–317.5	16.1–18.2	267.7–333.4

Supplementary Figures S3(A)-(B)
The actual numerical information of the *p*-values between each group is as shown in the table.

(A)	*p*-values		
	Left knee joint diameter	Mankin score	OARSI score
Sham vs. M	0.0017	<0.0001	<0.0001
Sham vs. M+(LD)LDL	0.3527	0.0353	0.0048
Sham vs. M+(HD)LDL	0.7561	0.1773	0.0747
Sham vs. M+(LD)HK-LDL	0.6745	0.0261	0.0346
Sham vs. M+(HD)HK-LDL	0.7252	>0.9999	>0.9999
M vs. M+(LD)LDL	0.2546	0.2267	>0.9999
M vs. M+(HD)LDL	0.0651	0.0444	0.1816
M vs. M+(LD)HK-LDL	0.089	0.2922	0.3482
M vs. M+(HD)HK-LDL	0.0737	0.001	0.0044
M+(LD)LDL vs. M+(HD)LDL	0.985	>0.9999	>0.9999
M+(LD)LDL vs. M+(LD)HK-LDL	0.9948	>0.9999	>0.9999
M+(LD)LDL vs. M+(HD)HK-LDL	0.9897	>0.9999	0.6462
M+(HD)LDL vs. M+(LD)HK-LDL	>0.9999	>0.9999	>0.9999
M+(HD)LDL vs. M+(HD)HK-LDL	>0.9999	>0.9999	>0.9999
M+(LD)HK-LDL vs. M+(HD)HK-LDL	>0.9999	>0.9999	>0.9999

(B)	*p*-values		
	MMP-1	MMP-3	MMP-13
Sham vs. M	0.3178	0.0117	0.0298
Sham vs. M+(LD)LDL	0.0033	0.0003	0.5715
Sham vs. M+(HD)LDL	0.0954	0.0073	0.2491
Sham vs. M+(LD)HK-LDL	0.1365	0.0051	0.8685
Sham vs. M+(HD)HK-LDL	0.1546	0.6476	0.7171
M vs. M+(LD)LDL	0.4102	0.8392	0.6384
M vs. M+(HD)LDL	0.9884	>0.9999	0.9246
M vs. M+(LD)HK-LDL	0.9973	0.9997	0.3224
M vs. M+(HD)HK-LDL	0.9986	0.3495	0.0005
M+(LD)LDL vs. M+(HD)LDL	0.792	0.9087	0.9923
M+(LD)LDL vs. M+(LD)HK-LDL	0.6956	0.9463	0.9948
M+(LD)LDL vs. M+(HD)HK-LDL	0.658	0.0287	0.0411
M+(HD)LDL vs. M+(LD)HK-LDL	>0.9999	>0.9999	0.879
M+(HD)LDL vs. M+(HD)HK-LDL	>0.9999	0.2646	0.0091
M+(LD)HK-LDL vs. M+(HD)HK-LDL	>0.9999	0.2106	0.1356

Supplementary Figures S3(C)-(D)
The actual numerical information of the *p*-values between each group is as shown in the table.

(C)

	p-values	
	PINP	CTX-I
Sham vs. M	0.9104	0.1322
Sham vs. M+(LD)LDL	0.2453	0.0614
Sham vs. M+(HD)LDL	0.9071	0.3752
Sham vs. M+(LD)HK-LDL	0.9293	0.089
Sham vs. M+(HD)HK-LDL	0.9996	0.3997
M vs. M+(LD)LDL	0.8261	0.9993
M vs. M+(HD)LDL	>0.9999	0.9918
M vs. M+(LD)HK-LDL	>0.9999	>0.9999
M vs. M+(HD)HK-LDL	0.9796	0.9886
M+(LD)LDL vs. M+(HD)LDL	0.8308	0.9372
M+(LD)LDL vs. M+(LD)HK-LDL	0.7958	>0.9999
M+(LD)LDL vs. M+(HD)HK-LDL	0.4003	0.9248
M+(HD)LDL vs. M+(LD)HK-LDL	>0.9999	0.9719
M+(HD)LDL vs. M+(HD)HK-LDL	0.9784	>0.9999
M+(LD)HK-LDL vs. M+(HD)HK-LDL	0.9862	0.9645

(D)

	p-values			
	TNF-α	IL-1β	IL-6	IL-10
Sham vs. M	0.8268	0.6951	0.6085	0.9989
Sham vs. M+(LD)LDL	<0.0001	0.5355	0.6425	0.9598
Sham vs. M+(HD)LDL	0.1497	0.1069	0.9968	0.9673
Sham vs. M+(LD)HK-LDL	0.9974	0.1749	0.9501	0.9992
Sham vs. M+(HD)HK-LDL	0.0835	0.2292	0.9315	0.3072
M vs. M+(LD)LDL	0.0022	0.9996	>0.9999	0.9975
M vs. M+(HD)LDL	0.796	0.8109	0.8713	0.846
M vs. M+(LD)HK-LDL	0.9727	0.9159	0.979	0.9757
M vs. M+(HD)HK-LDL	0.6349	0.9563	0.9869	0.1526
M+(LD)LDL vs. M+(HD)LDL	0.0678	0.9454	0.8927	0.5877
M+(LD)LDL vs. M+(LD)HK-LDL	0.0002	0.9859	0.9852	0.8398
M+(LD)LDL vs. M+(HD)HK-LDL	0.1241	0.9955	0.9912	0.0568
M+(HD)LDL vs. M+(LD)HK-LDL	0.34	0.9999	0.9985	0.9979
M+(HD)LDL vs. M+(HD)HK-LDL	0.9998	0.9986	0.9968	0.7794
M+(LD)HK-LDL vs. M+(HD)HK-LDL	0.2131	>0.9999	>0.9999	0.5122

Supplementary Figures S4
Effects of live or heat-killed LDL557 with low or high dose on the morphology of the MIA-induced OA rat's articular cartilage (H&E stain).

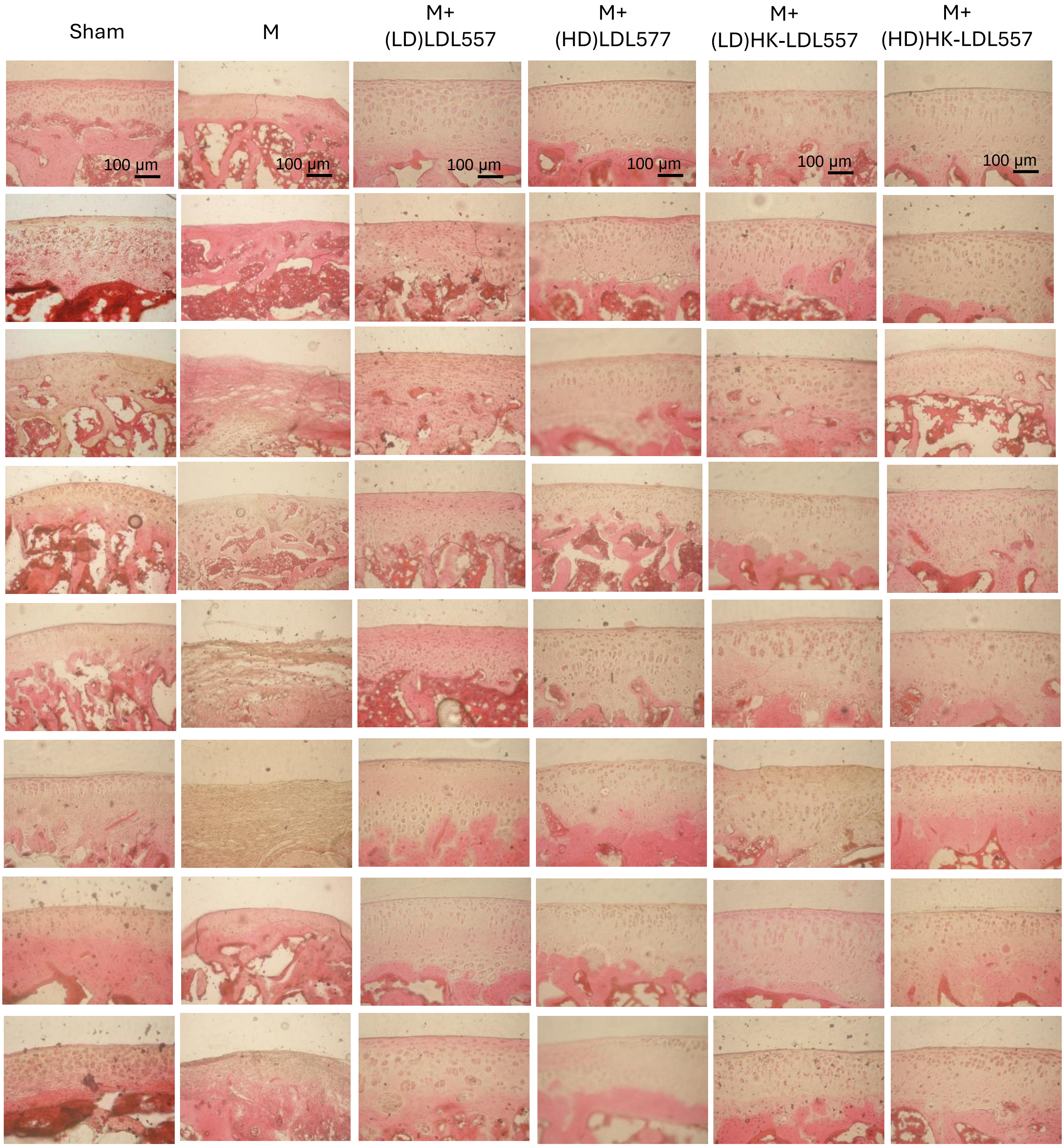

Supplementary Figures S5
Effects of live or heat-killed LDL557 with low or high dose on the histology of the MIA-induced OA rat's articular cartilage (safranin O/fast green).

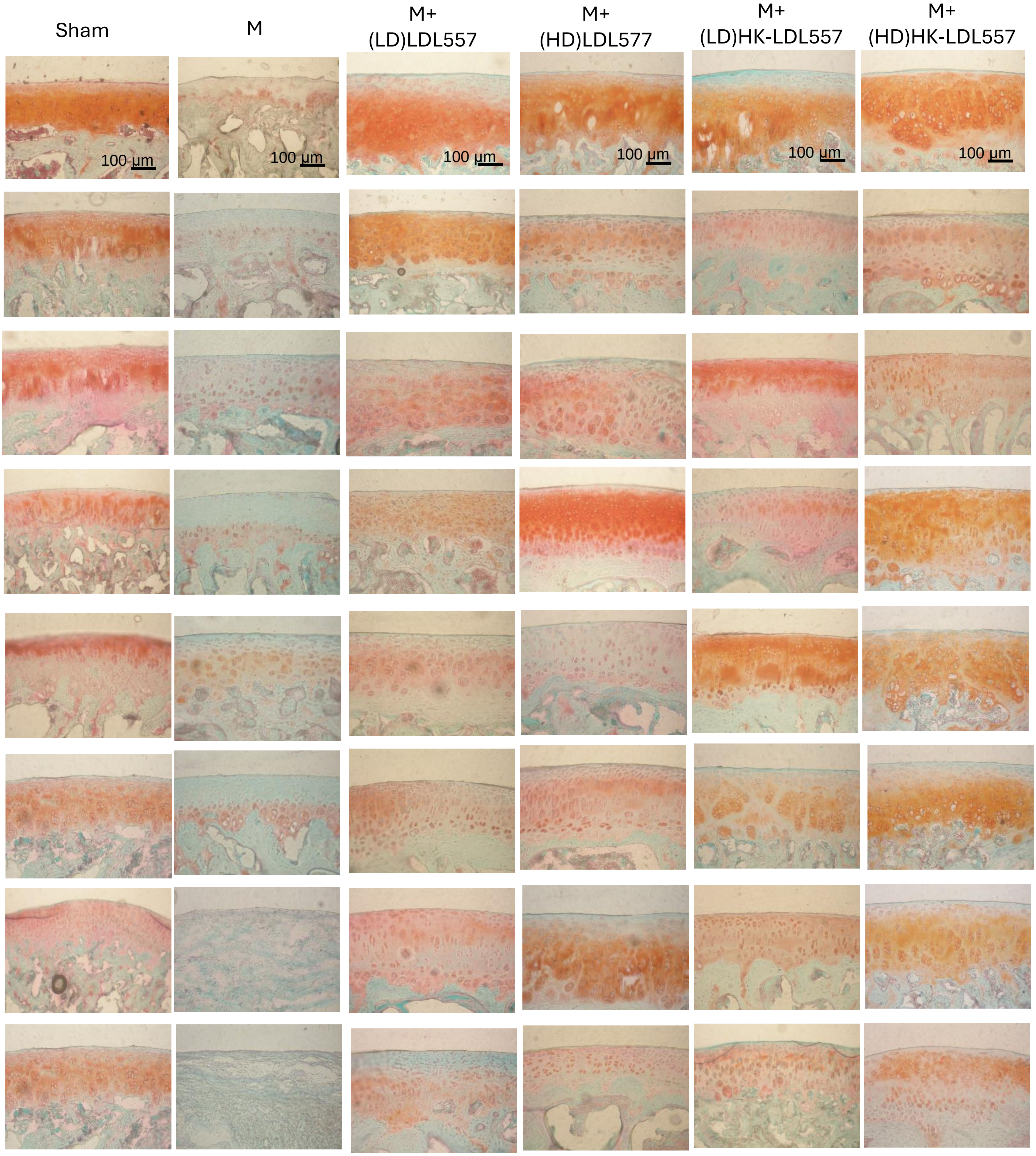

Supplement: Supplementary file 1 [file cimb-46-00530-s001.zip › cimb-3100766-supplementary.pdf]
